# “Corporate Digital Responsibility”: New corporate responsibilities in the digital age

**DOI:** 10.1007/s00550-020-00509-x

**Published:** 2021-02-01

**Authors:** Christina J. Herden, Ervin Alliu, André Cakici, Thibaut Cormier, Catherine Deguelle, Sahil Gambhir, Caleb Griffiths, Shrishti Gupta, Sahil R. Kamani, Yonca-Selda Kiratli, Máté Kispataki, Greta Lange, Leandro Moles de Matos, Laura Tripero Moreno, Hector Alain Betancourt Nunez, Venkata Pilla, Bairesh Raj, James Roe, Markus Skoda, Youye Song, Praveen Kumar Ummadi, Laura Marie Edinger-Schons

**Affiliations:** 1 Mannheim Business School, Mannheim, Germany; 2 Chair of Corporate Social Responsibility, University of Mannheim, Mannheim, Germany

**Keywords:** Corporate Digital Responsibility, Digitalization, Sustainability, Digital transformation, Digital age

## Abstract

Digitalization is leading to profound changes in our private and work lives. New technologies are pervasive and create opportunities for new business models and lifestyles. Recently, the term “Corporate Digital Responsibility” has been coined to summarize the emerging responsibilities of corporations relating to their digitalization-related impacts, risks, challenges, and opportunities. The paper at hand reviews the topic of CDR using a multi-step approach. First, results from an opinion poll of 509 US-based respondents are reported which illustrate the perceived opportunities and threats associated with the topic of digitalization, underlining the need for a strategic approach to CDR implementation. Second, existing uses and definitions of the CDR terminology are summarized and a definition of CDR is derived. Third, twenty important topics related to CDR are identified, summarized and categorized into three categories using the ESG (Environmental, Social, Governance) framework. Finally, results are discussed with regards to their theoretical and managerial contributions and a hands-on guide which companies can use to implement a suitable CDR strategy is presented.

## Introduction

Digitalization is leading to profound changes in our private and working lives by creating opportunities for innovative business models and lifestyles (Loebbecke and Picot 2015). As a consequence, many individuals and corporations have embraced the digital transformation (Hess et al. 2016). However, as with any massive disruption, the digital transformation involves both opportunities and threats. New technologies can enhance as well as impede energy efficiency and environmental impact (Herring and Roy 2007). Further, although technical innovations can lead to inclusion of many disadvantaged societal groups, unequal access to the digital world is already a reality (Bélanger and Carter 2009). Moreover, other examples of potentially negative consequences of the digital transformation include unemployment caused by automation and robotics or the danger of data breaches and cyber-attacks (Vial 2019). As a result, innovative technology brings with it new social issues and heightened responsibilities, especially for corporations (Vial 2019). These new responsibilities have recently been coined “Corporate Digital Responsibility” (CDR) (Thorun 2018).

CDR will likely become a differentiator for organizations, allowing them to gain and maintain stakeholder trust and competitive advantage (Koch and Windsperger 2017). Thus, it is high time for organizations to develop and implement a CDR strategy. The goal of such a strategy should be not only to prevent the potential negative consequences outlined above, but to also leverage the advantages of information communication technologies (ICTs) for the common good. Companies could, for example, engage in digital social innovation (DSI), which involves the development and implementation of innovative products, services, processes, business models and other innovative activities (in which digital technology plays a central role) that seek to address social or environmental problems (Digital Social Innovation 2015). Despite the growing number of digital solutions that aim to address a variety of social and environmental issues, existing DSI’s still do not provide the necessary solutions to tackle challenges related to the climate crisis, global water scarcity, species extinction, or global pandemics like Covid-19 (Digital Social Innovation 2015).

Currently, there are relatively few and widely differing definitions for the term “Corporate Digital Responsibility”. Chap. 3 provides an overview of existing definitions, conceptualizations, and uses of the term. They can broadly be summarized as a company’s emerging responsibilities related to their digitalization-related impacts, risks, challenges, and opportunities. The topic of CDR has very recently been addressed by Lobschat and colleagues (Lobschat et al. 2019) who argue that CDR should be considered separately from Corporate Social Responsibility (CSR) and propose that the basic conceptual constituents of CDR include four stakeholders (i.e., organizations, individual actors, institutional/governmental/legal actors, and artificial/technological actors) and four life cycle stages (i.e., the creation, operation, impact assessment, and refinement of technology) linked to digital technology and data. They further introduce a CDR framework that focuses on an organization’s CDR culture. While we appreciate their seminal conceptual work on the topic, the perspective of our current paper is different in that we regard CDR as an extension of CSR, comprising all levels of corporate responsibilities as defined in Carroll’s (1991) CSR pyramid and all domains of the Environmental, Social, Governance (ESG) framework.

We review the topic of CDR using a multi-step approach. First, we present results from an opinion poll of 509 US-based respondents that illustrate the thoughts and fears associated with the topic of digitalization. Second, we summarize existing uses and definitions of the CDR terminology and derive a practice-oriented definition of CDR. Third, we categorize important topics of CDR using the ESG framework. Fourth, we discuss our results with regard to their theoretical and managerial contributions and provide a hands-on implementation guide that companies can use to successfully develop and execute a CDR strategy.

## Perceived opportunities and threats of digitalization

The past technological transformations (e.g., industrialization) have had a major influence on shaping human societies (Kemp 2013). These new technologies resulted in several positive developments such as improvements in quality of life of citizens (Nelson and Lorence 1985) but were also accompanied by negative impacts such as environmental degradation (Cherniwchan 2012) or social inequalities (Stearns 2013). The emergence of the new digital era confronts today’s societies with a similar fate, presenting several opportunities as well as threats.

To gain a deeper understanding of the prevailing public perceptions of opportunities and threats of digitalization and to provide an initial overview of relevant topics that need to be addressed by corporations in their CDR strategy, an online survey was conducted amongst 509 US-based respondents.^1^ Based on the existing literature, a list of potential opportunities and threats was drafted and participants were asked to rate their agreement to the statements on 7‑point Likert scales (ranging from 1 = I do not agree at all, to 7 = I fully agree).

**Fig. 1:**
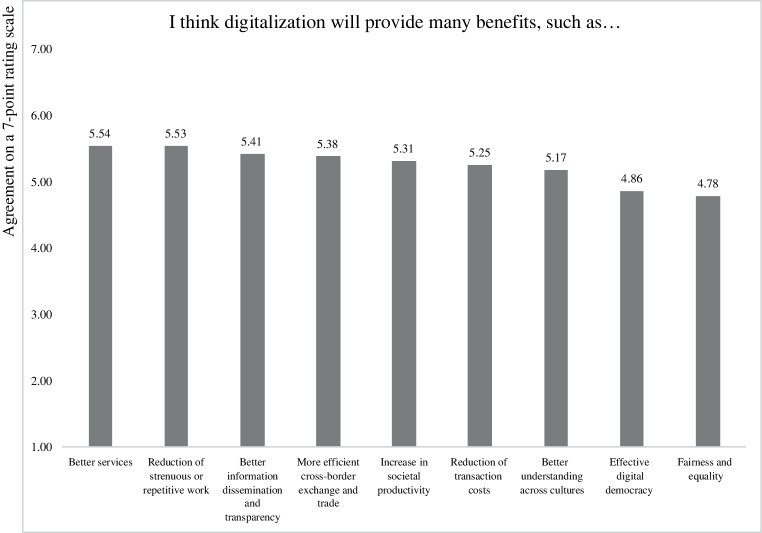
Perceived opportunities of digitalization

**Fig. 2:**
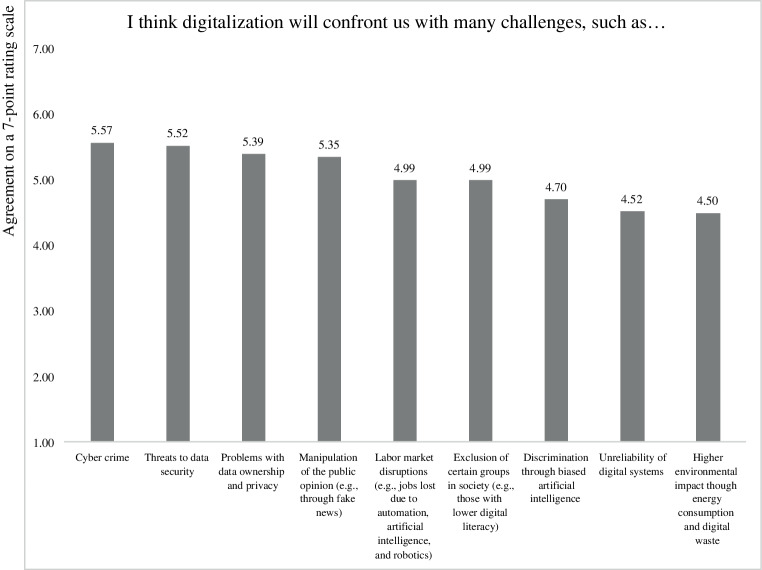
Perceived threats of digitalization

Figs. 1 and 2 summarize the mean responses to these items. The potential opportunities of digitalization that received the most agreement by respondents included “better services”, “reduction of strenuous or repetitive work for humans”, and “better information dissemination and transparency”. In contrast, the opportunities that respondents agreed with the least (even though the mean values for these items were still above the scale midpoint) included “fairness and equality”, “effective digital democracy” and “better understanding across cultures”. Regarding the potential threats of digitalization, the items that received the highest agreement among respondents included “cyber-crime”, “threats to data security”, and “problems with data ownership and privacy”. The items that received the least agreement (but were still above the scale midpoint) included “higher environmental impact through energy consumption and digital waste”, “unreliability of digital systems”, and “discrimination through biased artificial intelligence”.

**Fig. 3:**
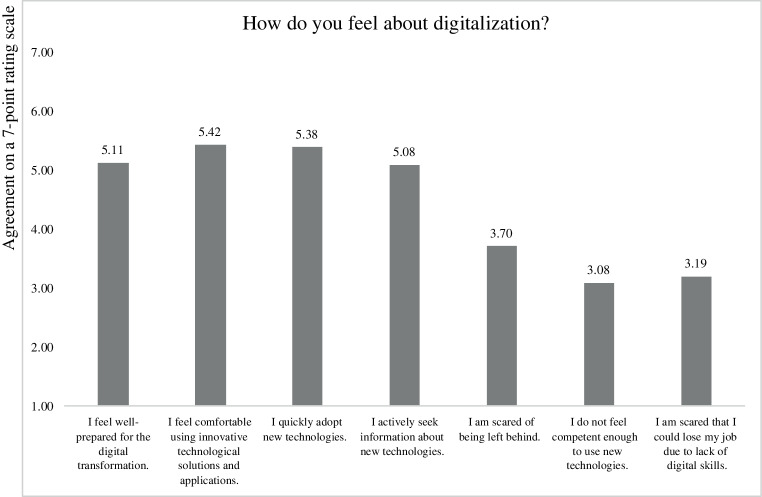
Feelings regarding preparedness for digitalization

In addition, we also included items that addressed whether respondents felt well-prepared for digitalization. Fig. 3 summarizes these responses. It is interesting to note that 30% of respondents rather agreed, agreed, or even strongly agreed to the item “I am scared that I could lose my job due to lack of digital skills”. Furthermore, when asked whether respondents felt that their employer is well-prepared for digitalization, 45.5% of respondents disagreed, 17.1% neither agreed nor disagreed, and 37.3% agreed to the statement “Our company has failed to embrace the digital transformation”. These preliminary findings reveal an opportunity for companies to tackle the threats and embrace the opportunities of digitalization in the corporate setting by implementing a CDR strategy.

The data generated by the survey also provided comprehensive insights through answers in open text boxes which we utilized to source topics relating to CDR. Table 1 summarizes the most important opportunities and threats mentioned in the survey responses which we juxtaposed and complemented with insights from existing literature.

**Table 1:** Opportunities and Threats of Digitalization

*Potential Opportunities*	
Social Inclusion	The rate of information dissemination in today’s digital era has enabled more efficient and comprehensive interactions between people (Tsatsou 2011) and has allowed for faster exchange of culture and knowledge between those from different backgrounds and paradigms (Andrade and Doolin 2016)
Efficiency in public service delivery	Open technologies are impacting the design and delivery of public utilities and systems (Kirov 2017)—channels of public service delivery are becoming faster, cheaper and more efficient through digitalization, and interactions between citizens and governments are enhanced through e‑government initiatives (Kirov 2017)
Operational efficiency and ease of knowledge acquisition	Through automation of business operations, transaction costs are significantly reduced, and, combined with the ease at which people can acquire new information, has led to an increase in societal productivity (UNCTAD 2017, 2018, 2019)
Business opportunities and employment generation	Digitalization enables alternative financing opportunities for entrepreneurs such as crowdfunding and reduces barriers of expansion, leading to a more efficient cross-border exchange of goods and services (Bouncken et al. 2015). Digital technologies such as cloud solutions and peer-to-peer collaboration allow companies to shift supporting business processes to other parts of the world with cheaper labor supply (Kahl et al. 2017)
*Potential Threats*	
Workforce disruption	Digitalization has led to a decrease in demand for low level repetitive jobs that have been automated by digital technologies, and the emergence of collaborate business models is also redefining new skill requirements for the labor market and the educational curriculum (McKinsey Global Institute 2017)
Cybercrimes	Digitalization has increased the vulnerability of individuals and business to malicious attacks (Marcum and Higgins 2019), which also target online communities that may consists of millions of individuals (Marcum and Higgins 2019)
Data privacy issues	The ease of access to personal information has created a new set of privacy issues which were not prominent before the digital era (Elahi 2009), resulting in an evolution of policies that govern data privacy concerns of individuals (Elahi 2009)
Social engineering and media manipulation	The susceptibility to influence and manipulation has increased in size and breadth as a result of digitalization and new capabilities now enable basic human behaviors to easily be exploited remotely by malicious attackers to achieve an economic or political agenda (Marwick and Lewis 2017)

## Defining corporate digital responsibility

There is currently not yet a consensus about the definition of the term, “Corporate Digital Responsibility”. Consequently, the term CDR is often used in a variety of different ways. Table 2 summarizes some existing definitions, conceptualizations, and uses of this term.

**Table 2:** Existing definitions, conceptualizations, and uses of the term CDR

Definition	Source
“The set of shared values and norms guiding an organization’s operations with respect to the creation and operation of digital technology and data.”	Lobschat et al. (2019)
“Expanding the remit of CSR to address the impact of the digital tools and environments that businesses operate in.”	Ampofo (2016)
“CDR is about recognizing that the organizations driving forward the advancement of technology, and those that leverage technology to engage and provide services to the citizen, have a responsibility to do so in a manner that is fundamentally leading us toward a positive future.” “A CSR strategy that spans the breadth of technology’s impact on society.”	Joynson (2018)
“Corporate Digital Responsibility is about protecting people’s rights around data (in line with regulation), about ensuring that trust is maintained because they see that products and services save them personal time, help them with their health and ageing, and protect them from less acceptable or threatening uses of those same technologies.”	Price (2018)
“Corporate Digital Responsibility (CDR) refers to corporate responsibility in the digital society.”	CSR News (2018)
“Corporate Digital Responsibility is an understanding of corporate responsibility in and for a digital society. It involves a regulated and a voluntary level: on the one hand, the observance of relevant laws or directives, on the other hand, the exercise of a voluntary responsibility in shaping the digital society.”	politik digital e. V. (2018)
“A Corporate Digital Responsibility (CDR) complements corporate responsibility and partially re-thinks it as companies need to think about the societal impact of digital products and services as they evolve and ensure that they are compatible with our value standards.”	Andersen (2018)
“Corporate digital responsibility is a voluntary commitment. It starts with the need to conform to legal requirements and standards—for handling customer data, confidential, intellectual property and so on—but it also extends to wider ethical considerations and the fundamental values that an organization operates by.”	Driesens (2017)

Based on our review of existing definitions of CDR listed above and our own conceptual work on the topic, we propose the following definition of CDR:

Corporate Digital Responsibility is an extension of a firm’s responsibilities which takes into account the ethical opportunities and challenges of digitalization.

**Fig. 4:**
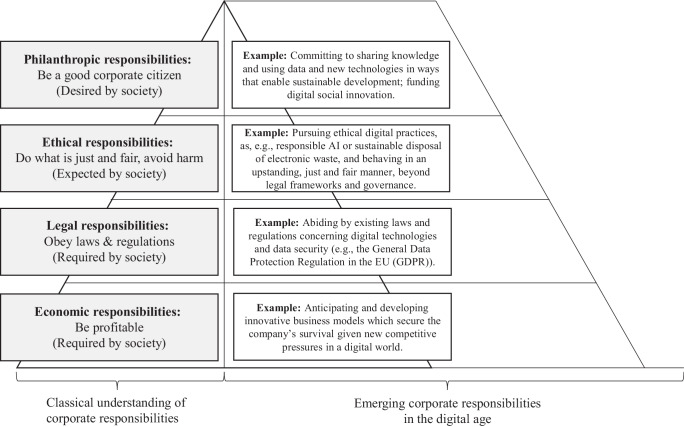
The CDR pyramid

Similar to the responsibilities proposed in the CSR pyramid developed by Carroll in the 1990s (Carroll 1991), we propose that corporate digital responsibilities also encompass four different levels (i.e., economic, legal, ethical, and discretionary). First, at the economic level, it is important for firms to find innovative business models which secure their competitive advantage given new competitive pressures in a digital world (Koch and Windsperger 2017). Second, at the legal level, it is mandatory for companies to abide by existing laws and regulations concerning digital technologies and data security (e.g., the General Data Protection Regulation in the EU (GDPR)) (Voigt and Busche 2017). Companies may not be able to manage this on their own and may need support from the government, academics, or consultancies (Sadiq and Governatori 2014). Microsoft, for example, is working together with government authorities to better grapple with the legal issues surrounding advancing face recognition technology (Smith 2018). On the third level, ethical responsibilities, meaning avoiding harm and providing benefits for stakeholders by using digital technologies, it is becoming increasingly important for companies to pursue ethical practices, and behave in an upstanding, just and fair manner, beyond legal frameworks and governance, thereby fulfilling their stakeholders’ heightened expectations (Carroll 1991). In fact, some firms, such as SAP, have assembled ethical advisory panels for topics related to artificial intelligence (AI) (SAP 2018). Finally, at the fourth level, discretionary responsibility, firms may include philanthropic efforts that are beneficial to society by, for instance, committing to sharing knowledge and using data and new technologies in ways that enable sustainable development (Stempeck 2014). They may also decide to contribute to society by supporting funding schemes for digital social innovation, such as ones in the social entrepreneurship sector (Hackenberg and Empter 2011). Fig. 4 provides an illustration of the classical understanding of corporate responsibilities and examples of the emerging new topics in the digital age on the four levels proposed by Carroll (1991).

## Relevant topics for CDR

A first step in better understanding the concept of CDR is to establish which topics fall under its scope and to determine how these topics can be classified in a meaningful way. A well-established framework that is most commonly used to categorize topics related to CSR is the ESG model (Kocmanová and Šimberová 2014). Thereby, the ESG criteria represent the three main factors related to a firm’s ethical impacts and sustainable practices that offer an investor potential long-term performance advantages (Friede et al. 2015).

We propose that the ESG framework is suitable to categorize topics related to CDR. Considering the complex and evolving field of CDR, topics within categories may overlap and be interlinked when such a framework is applied (Hayat and Orsagh 2015).

What follows is a list of the currently most relevant topics related to CDR categorized according the ESG domains (see also Table 3). The list is the culmination of a workshop on CDR in which we engaged in an intensive search of academic and non-academic sources on the topic. It was created by a comprehensive review of existing literature and by applying relevant CSR-related principles and concepts to the increasingly important use cases in the current and future digital landscape. This list is by no means exhaustive and may likely evolve in the future, especially given the rapidly changing nature of the digital age.

**Table 3:** CDR topics categorized according to the ESG framework

Environmental	Social	Governance
1. Energy and carbon footprint	1. Digital cohesion	1. Reliability of system
2. Digital waste	2. Digital influence	2. Data transparency
	3. Digital well-being	3. Data collection and storage
	4. Digital empowerment	4. Data ownership and privacy
	5. Socially compatible automation	5. Data responsibility and stewardship
	6. Unbiased AI	6. Data security
	7. Digital self	7. Data usage and accessibility
	8. Digital inclusion	8. Robot ethics
	9. Digital surveillance	
	10. Digital freedom	

### Environmental CDR

#### Energy and carbon footprint

The use of Information and Communication Technology (ICT) in society is growing continuously driven by the development of new products and solutions (Malmodin et al. 2014). Hilty and Bieser (2017) classify environmental effects of ICT into direct and indirect effects. The emission of greenhouse gases (carbon footprint) is a direct environmental effect and encompasses carbon footprint changes of the entire life cycle of ICT hardware starting from the production (e.g. mining of resources, energy consumption), the operation (e.g. energy consumption), to the disposal (e.g. recycling) of ICT devices and infrastructure. Indirect effects account for greenhouse gas changes of other sectors applying ICT technology. Based on research by Hilty and Bieser (2017) with a focus on Switzerland, the environmental footprint is strongly caused by the production of ICT hardware.

The Information and Communication Technology (ICT) sector was estimated to represent between 5–9% of total electricity consumption in 2018 and it is expected to rise to 20% in 2030 (Enerdata 2018). This percentage will likely keep growing due to the greater accessibility of digital platforms to the world population and the increase in individual use of digital technologies, for instance during the recent global Covid-19 pandemic (Global e‑Sustainability Initiative GeSI 2019; Beech 2020). On the other hand, ICT carbon emissions as a percentage of global emissions are expected to decrease to 1.97% in 2030, compared to 2.3% in 2020 (Global e‑Sustainability Initiative GeSI 2019). Over the last years, ICT companies have had a greater commitment to power their infrastructure (e.g., data centers and communication networks) with renewable energy sources, mainly because of the increasing cost competitiveness of renewable energy, the customers’ rising interest in having their digital structure powered by clean energy, and the associated goal of ICT companies to improve their brand identity (Ahmed et al. 2017).

#### Digital waste

The corporate sector has been the earliest consumer of ICT and currently holds a sizable share of total installed ICT equipment (Baldé et al. 2017). The installment of new operating systems or better performing applications makes electrical and electronic equipment obsolete which is consequently passed on to dismantlers or recyclers (United Nations Environmental Programme 2007). The European Commission has recently adopted a new Circular Economy Action Plan that aims to reduce the EU’s consumption footprint and double its use rate of circular material by working together with businesses to create a basis for sustainable products (European Commission 2020). As a result, some companies are becoming increasingly more vigilant regarding the replenishment and recycling of their ICT equipment. Most of the materials contained in this equipment are recyclable and reusable, and may even include precious metals such as gold, platinum, or rare elements (i.e., tantalum, lanthanum, or neodymium) (Manhart et al. 2016). For example, there is 50 times more gold in a ton of electro-technical cards than in one ton of ore (Manhart et al. 2016). Other materials are dangerous for the environment and human health (i.e., lead, bromine, arsenic, chlorine, mercury, cadmium, etc.) and must be treated (Manhart et al. 2016). In many industrialized countries, improved dismantling can contribute to increased material recovery. Unfortunately, most industrialized and developing countries still lack the capacity for environmentally sound recycling (Chi et al. 2011). As a consequence, large amounts of digital waste enter informal recycling processes with severe environmental and social consequences. In order to help tackle the electronic waste issue, it is recommended that corporations implement and enforce responsible recycling practices, refrain from selling waste to developing countries, and support projects that allow customers to send back their old devices. For instance, Europe’s largest non-profit IT company, AfB, employs workers with disabilities to refurbish and resell IT and mobile equipment from large companies and public institutions (AfB 2019).

### Social CDR

#### Digital cohesion

The world is crossing into the phase of digital disruption, an era of new technologies and business models that changed user expectations and the value proposition of existing goods and services (Zaki 2019). As users grow accustomed to digital products and services that reduce the complexity of daily lives, their expectations that technology should adapt to their daily needs and behaviors will likely grow. Such growth in user expectations and latest advancements in technology will change the way in which humans interact with technology (Cascio and Montealegre 2016). This phase is called digital cohesion, an era in which multiple applications connect and self-assemble to provide autonomous systems and deliver predictive services that continually adapt to personal behaviors, improving human lives (Singh Batth et al. 2018). For example, such services can be important in healthcare—wearable biosensor technology can provide accurate and reliable real-time sensing of physiological information that can help improve the daily lives of patients (Kim et al. 2019). To make digital cohesion a reality, an ecosystem has to be formed, with technology as the backbone, in which technology companies, industry alliances, government regulations and AI come together to deliver high performing interoperable services.

#### Digital influence

Digital influence refers to the power to effect a change in opinion or behaviors of other online users in a measurable way. This ability is usually multiplied through social networks or other online platforms (Anderson 2017; Hayta 2013; Valenzuela 2013). People that possess the power to exert digital influence have been coined influencers (Kiss and Martin 2008; Uzunoğlu and Misci Kip 2014). Though this term most commonly refers to people with a large group of followers who receive payment for promoting a product, influencers may also be real-life friends or online reviewers who lead a consumer to purchase a certain product through positive reviews (Jiménez and Mendoza 2013). Digital influence is measured in reach (i.e., more popular topics will reach more people), relevance (i.e., the alignment of the influence to current public interests) and resonance (i.e., the duration of time for which a thread or its ripple effect remains present) (Robinson 2017). As most influencers likely have varied understandings of their responsibility in exerting influence, it is important that the operators of online platforms exercise responsible oversight. This can be done by preventing misuse of unconsented data, providing clarification on and raising awareness of the power of their platform, and creating clear and transparent rules for freedom of speech, leading to increased transparency and governance over influencers’ content.

#### Digital well-being

Digital well-being refers to a mature and appropriate handling of digital media, as well as an increased awareness of the potential addictions that can result from usage and the connection between digital behavior and mental disorders (Scott et al. 2017). The more tasks and aspects of everyday life move online to web and smartphone apps, the more time is spent looking at screens. This results in a heightened urge or even a reflex to continuously check cell phones and online profiles for updates and news, which can ultimately lead to addictive behaviors and the overuse of screen time (De-Sola Gutierrez et al. 2016). This increasing digitalization can lead to disconnection and isolation from the real world and may affect mental health (Hayes et al. 2016). Therefore, it is of utmost importance that corporations support their users in finding a right balance for their usage of digital products by encouraging responsible use and ensuring that technology enables users to stay connected to the real world.

#### Digital empowerment

Digital Empowerment can range from an increased number of economic opportunities to enabling more social contact for otherwise isolated individuals (Mäkinen 2006). This is especially relevant during times of quarantine and social distancing experienced during the global Covid-19 crisis (Wilder-Smith and Friedman 2020). A digital presence can not only allow individuals to work from home, but also allow companies to expand the reach of their offerings and enable the establishment of a business at a much lower capital investment than with a brick-and-mortar office. For older adults, the connection to the online world provides the opportunity to increase social contact and decrease loneliness (Cotten et al. 2013).

With the increase in online participation, companies and individuals alike need to develop a way to cope with the responsibility of having access to a wealth of data and information. Additionally, “the ever-evolving nature of technology [also] means that individuals need ever increasing levels of digital literacy to maintain their sense of inclusion” (Hill et al. 2015, p. 415), which can be especially difficult for those individuals who could benefit the most from increased social interaction online. Corporations could help address this by becoming more democratic and asking for the opinions of their employees with the help of digital tools (Burnett and Lisk 2019). They could also build platforms that encourage social exchange and allow for flexible working hours while ensuring that work-life balance is maintained (Kossek et al. 2014). In addition, digital empowerment can be nurtured by raising awareness and providing education. Creating well-educated digital citizens will lead to a more ethical, safe, and responsible digital environment (Heick 2018).

#### Socially compatible automation

With the rise of automated systems, some jobs might be automated leaving humans who were previously responsible for these jobs unemployed (Arntz et al. 2017). Preparing more students and workers for careers in well-paying new-collar jobs using modernized education systems will encourage them to acquire sought-after skills and competencies rather than pursue specific academic degrees (Chamorro-Premuzic and Frankiewicz 2019). Publishing such policies on retraining and redeploying employees that are subject to automation systems will ensure productivity, economic growth, and job creation. Organizations should ensure that new roles are created with new emerging technologies and that humans retain value in the future. It can be seen as their responsibility to make employees prepared in advance for such business shifts and technological changes (Bean 2017).

#### Unbiased AI

Today, the term artificial intelligence (AI) is increasingly used to describe machines that can mimic human functions such as learning and problem solving (Rastogi 2017). As society debates the implications of AI systems, corporates must ensure AI systems treat everyone of all abilities and disabilities fair and without discrimination so that they reflect the diversity of the world in which we live (Dignum 2018). They must also ensure that people understand the algorithms used in development of AI and how it came to a given conclusion or recommendation, thus empowering people through digital inclusion. In addition, corporations must constantly monitor the self-learning AI tools and be accountable for any misbehavior. They must also work with policymakers, governments, and clients to prepare the workforce with the skills needed to work effectively in partnership with AI systems (Dignum 2018). One example of a recent AI ethics initiative is the CDR Initiative of the Federal Ministry of Justice in Germany which, together with Telefónica Deutschland and other well-known German companies, set up a process for the development of CDR guidelines (BMJV 2019). In this context, Telefónica Deutschland has published ethical principles for the responsible and morally legitimate use of AI, which is crucial in its daily business (Telefónica Deutschland 2019).

#### Digital self

Digital self can be defined as the evolution of the “extended self” (Belk 2013). The three dimensions of the “extended-self” (i.e., body, internal processes, ideas and experiences, and the persons, places, and things to which one feels attached), have changed after decades of human interaction with multiple digital platforms (Belk 2013). From the early stages of e‑mail and instant messaging to the modern times of multiple social media outlets interacting at the same time, the information available online about any physical individual has increased significantly and has become more sensitive and difficult to trace and to protect (Chamorro-Premuzic 2015). Accordingly, the risk and the benefits associated with owning and managing a digital persona have increased in number and complexity. Considering the level of importance that digital media has reached in our current society, governments should create a new framework for safer and more efficient interactions between all relevant stakeholders in the digital world. Corporations who own the digital media should adhere to this framework and perform research on the possible transformation of different personas that may result from the use of their digital products (and release the results publicly before releasing their product into the market). Similarly, the process for handling data from deceased personas also needs to be addressed by companies.

#### Digital inclusion

Digital inclusion can be defined as the availability and accessibility of digital technologies (hardware, software and new frontiers such as the cloud) to users (European Commission 2019). This concept encompasses the roll out of technologies in both the developing and developed world (International Telecommunication Union (ITU) 2019). Provision of digital services should not discriminate between users or prevent marginalized or unserved/underserved users from completing basic activities. A pertinent example is a growing trend towards the provision of services exclusively online such as company job application processes and government services like applications for school loans and drivers licenses in the United Kingdom (UK) (Asthana and McVeigh 2010). The Free Software Foundation has identified six major threats to user’s freedom that are increased when digital inclusion is pursued without consideration for human rights: surveillance, censorship, proprietary software, restricted formats, software as a service and copyright infringement (Stallmann 2010). Further, digital inclusion is increasingly emerging as a direct “international development practice”, as non-governmental organizations (NGOs) and foreign aid programs seek to provide unserved populations with access to the Internet and Internet-based services. Dolan (2018) contends that investments in digital inclusion must cultivate trust amongst users in order for the Internet to remain a positive force for society; restoring the erosion of trust in digital mediums that has been driven by increasing misinformation, government and corporate surveillance techniques and cyber-crime.

#### Digital surveillance

Surveillance historically defined as “the focused, systematic, and routine attention to personal details for purposes of influence, management, protection, and direction” (Lyon 2007, p. 14) is now not just focused, systematic or routine but ubiquitous in this digital age. Increasingly, surveillance does not seem an activity undertaken for simple “influence, management, protection or direction” (Lyon 2007, p. 14), but instead seems to be much more, constituting the core security strategy of many nation-states and the core business model for the largest Internet firms, credit cards companies, and advertisers (Lyon 2007). Surveillance designed to feed the systems of industry, agriculture, public health, military, and education is getting better all the time, management and processing of such information has become an essential component for these systems. Corporations must ensure that new advances in the technology of encryption, usability and open protocols have the potential to offer powerful protection to the common user in the near future before surveillance invades into more aspects of social life (Zuboff 2018).

#### Digital freedom

Freedom is defined as “the condition or right of being able or allowed to do, say, think, etc. whatever you want to, without being controlled or limited” (Cambridge Dictionary 2020). This is logically extendable to include those same conditions or rights exercised in and through digital mediums. Whilst, under international law, ultimate responsibility for the protection of human rights online rests with states, users of products and services based on Internet infrastructure generally engage directly with companies (Brown and Korff 2016). A growing responsibility for supporting digital freedom therefore rests with corporate decision makers as well as government decision makers. The Global Network Initiative (GNI), an initiative that provides direction and guidance to the ICT industry on the global protection and advancement of “Freedom of Expression” and “Privacy” as human rights, is a positive example of industry attempting to manage both the internal and external pressures that conflict with digital freedom in a structured and accountable manner (Global Network Initiative 2017). Corporations must guarantee the data and privacy of their users and if they are unable to, they must commit to informing their users about potential third parties who may have access to their data (e.g. governments, etc.) (Global Network Initiative 2017).

### Governance CDR

#### Reliability of systems

In today’s information age, the world is producing more data than ever before and the usage of data centers to store data is growing continuously (Moorhead 2018). Significant investments in data centers have been witnessed by colocation, cloud, and telecommunication service providers (ReportLinker 2020). Data centers are becoming larger not just because of the increase in the amount of new data generated each year but also because of agglomeration of many small data centers into massive data centers to facilitate cost-cutting, energy efficiency, and ease of management. The metric of their reliability is the mean time to data loss (MTTDL) (Venkatesan and Iliadis 2012). As the dependency on data centers increases, assuring the reliability of these systems becomes more critical. The loss of access to a data center for less than a minute could mean significant financial losses for corporations. Thus, corporates should ensure not only that their own data storage systems are reliable but also that their outsourced data storage systems providers are selected, monitored, and managed in such a way that ensures reliability of the system at a holistic level.

#### Data transparency

In the context of telecommunications, data transparency means that the input and output data streams to and from a communication system have the exact same bit sequence (encyclopedia.com 2020). This is desirable so that users are not aware of the data processing being conducted, making complex systems easier to use. In the context of businesses, transparency is understood as the lack of hidden agendas and conditions, and a minimum degree of disclosure to which agreements, dealings, practices, and transactions are open to all for verification (BusinessDictionary 2020). In the context of CDR, both definitions have to be merged. Digital systems should be as simple as possible to use, while also being open for auditing by regulatory entities and, ideally, also by users. However, when the business definition is applied to the telecommunication context, it becomes difficult to reconstruct which lines of programming code perform a specific action. Programmers are able to hide the actual compiled code, and new programming techniques such as machine learning create code automatically that is complex for humans to read and understand (Burrell 2016).

#### Data collection and storage

While data collection methods vary by discipline, the emphasis on ensuring accurate collection remains the same (Northern Illinois University 2005). Corporates need to abide by the laws and regulations of the jurisdictions they operate in (Voigt and Busche 2017). As a consequence, they will need to put in place systems that conform to data protection requirements in their respective countries. As part of this, parties that get in contact with a digital touchpoint of a corporation should be provided with information about the data collection policy of the company and their consent should be obtained in order to proceed with the digital interaction (Voigt and Busche 2017). Different types of data storage play different roles in a computing environment (Wang et al. 2010). In addition to forms of local data storage, there are now new options for remote data storage, such as cloud computing, that can revolutionize the ways that users access data (Wang et al. 2010). The role of corporates is to conform to legal requirements of the jurisdiction. In case of the European Union (EU) and General Data Protection Regulation (GDPR), these include, but are not limited to, information provided on and consent to be obtained regarding the data storage policy of the corporation (Voigt and Busche 2017).

#### Data ownership and privacy

Data ownership is defined as the legal right to possess and control over data (Cattaneo et al. 2016; Hofheinz and Osimo 2017). Data owners have the right to access, modify, use and reuse data (Hofheinz and Osimo 2017). Data privacy or information privacy is concerned with the collection, protection, and dissemination of data (Barker et al. 2009). Because of the complexity of the digital word, it is difficult to determine who owns specific information (Hofheinz and Osimo 2017). Is the owner the one that creates the data or the one that it refers to? For example, when a user stores a personal photo in the cloud free of charge, does the company have any rights over this data? Corporations may take advantage due to their privacy policies, leaving consumers in an adverse position, not knowing how to protect their own privacy online (Isaak and Hanna 2018). By having clearer ownership and privacy policies that any user can understand, user-company relations can be improved, and unnecessary litigation can be avoided (Isaak and Hanna 2018).

#### Data responsibility and stewardship

It is essential that businesses collect personal data to develop more personalized services, drive next generation products and unlock new markets using new technologies (Boudet et al. 2019). However, organizations that collect, store, manage or process data have an obligation to handle it responsibly (Cooper and LaSalle 2016). They should act as stewards by taking the trust seriously and being responsible and accountable to data owners by ensuring effective control and use of data assets (Cooper and LaSalle 2016). Data stewardship addresses all phases of data lifecycle from data asset conception, creation, description and preservation to accessibility, reuse and beyond (Cooper and LaSalle 2016). As with businesses that collect data, the people who develop and deploy AI are also responsible for how they work (Mainzer 2019).

#### Data security

Due to the proliferation of digital services that require personal information to work and the subsequent increase in the devices that enable users to access these services, the amount of information that navigates on a daily basis and that is stored in servers all over the world is immense (Spiekermann et al. 2015). Through the lens of CDR, the interaction between corporations and its stakeholders is crucial to define and ensure data security (Lal Bhasin 2006). Companies should focus on collecting and storing the minimum information necessary to deliver a specific service while guaranteeing secure access and manipulation of this data (Cooper and LaSalle 2016). In addition, users must become increasingly aware of the value of their personal data and should decide carefully what information they share and with whom. To ensure continued access to user data, companies need to establish a sense of trust in their customers (Cooper and LaSalle 2016). To do this, data security must become a key priority within a company via increased transparency of data practices, effective security measurements, internal data security regulations, continuous review of the existing technical infrastructure, and appropriate training of employees (Cooper and LaSalle 2016).

#### Data usage and accessibility

In the current age, it is essential that individuals who need to have access to data have it when they require it. In addition, free movement of data across borders is essential to some industries in order to provide better insights and to grow the economy (Hofheinz and Osimo 2017). Owners of the data, not governments, should determine how their data is used, where their data is stored and how it is processed (Cattaneo et al. 2016). Corporations should use data only for the purposes which they claim to use it for, and companies who provide cloud data centers should offer these in different countries around the world to give data owners the flexibility to decide where they would prefer to store and process their data (Hon 2017). If a European customer, for example, purchases a cloud service from an American company, they may not wish to send their data to the United States. Thus, the cloud provider should give customers the option of storing the data in their local country, which means data centers must be available in multiple geographic locations. Furthermore, digital trade agreements must be mandated between the involved parties in order to enable and facilitate the cross-border flow of data and limit data localization requirements (Hill 2014).

#### Robot ethics

Today, robots assist human beings by performing jobs that are dangerous, distant, dirty, dull, or repetitive (Marr 2019). They are widely used in the workplace, manufacturing, assembly and packing, transport, earth and space exploration, surgery, weaponry, laboratory research, and mass production of consumer and industrial goods (Bellis 2019). Although autonomous service robots clearly hold tremendous advantages in many industries, use of such robots in healthcare or dangerous environments such as war zones are problematic and raise ethical challenges and issues (Lin et al. 2009; Sharkey and Sharkey 2012). The challenges could be legal such as unclear responsibility (whom to blame in case of improper conduct) or societal such as counter tactics in asymmetric wars (leading to different and uncertain ways of fighting a war) (Lin et al. 2009). To overcome such challenges, corporations when creating the robots must abide by the laws of robotics, a set of rules and principles that are intended to work as an initial framework to underpin the behavior of those robots designed to have a degree of autonomy (Murphy and Woods 2009). A consortium must be formed with experts from several disciplines, whose role it is to adjust these laws to fit new problems arising from advancements in robotics (Murphy and Woods 2009).

An overview of the above outlined topics and their categorization is provided in Table 3 below.

## Discussion

### Theoretical implications

Based on existing uses and definitions of CDR, we developed a practitioner-oriented definition which regards CDR as an extension of the responsibilities of companies, corresponding to the levels defined by Carroll (1991). According to this definition, CDR comprises topics from all domains of the ESG framework. Therefore, we added a novel perspective to the discussion around how CDR relates to CSR. To provide an overview of these CDR topics, twenty CDR-related issues were identified based on existing literature and the preliminary survey and were classified according to the three components of the ESG framework. Our results expand on existing knowledge of CDR by clearly articulating how CDR relates to the general responsibilities of companies (i.e., the economic, legal, ethical and discretionary dimensions) and by discussing which new topics might arise in these dimensions due to emerging new technologies. We further show that the ESG framework is a suitable categorization for summarizing the various CDR dimensions.

### Managerial implications

**Fig. 5:**
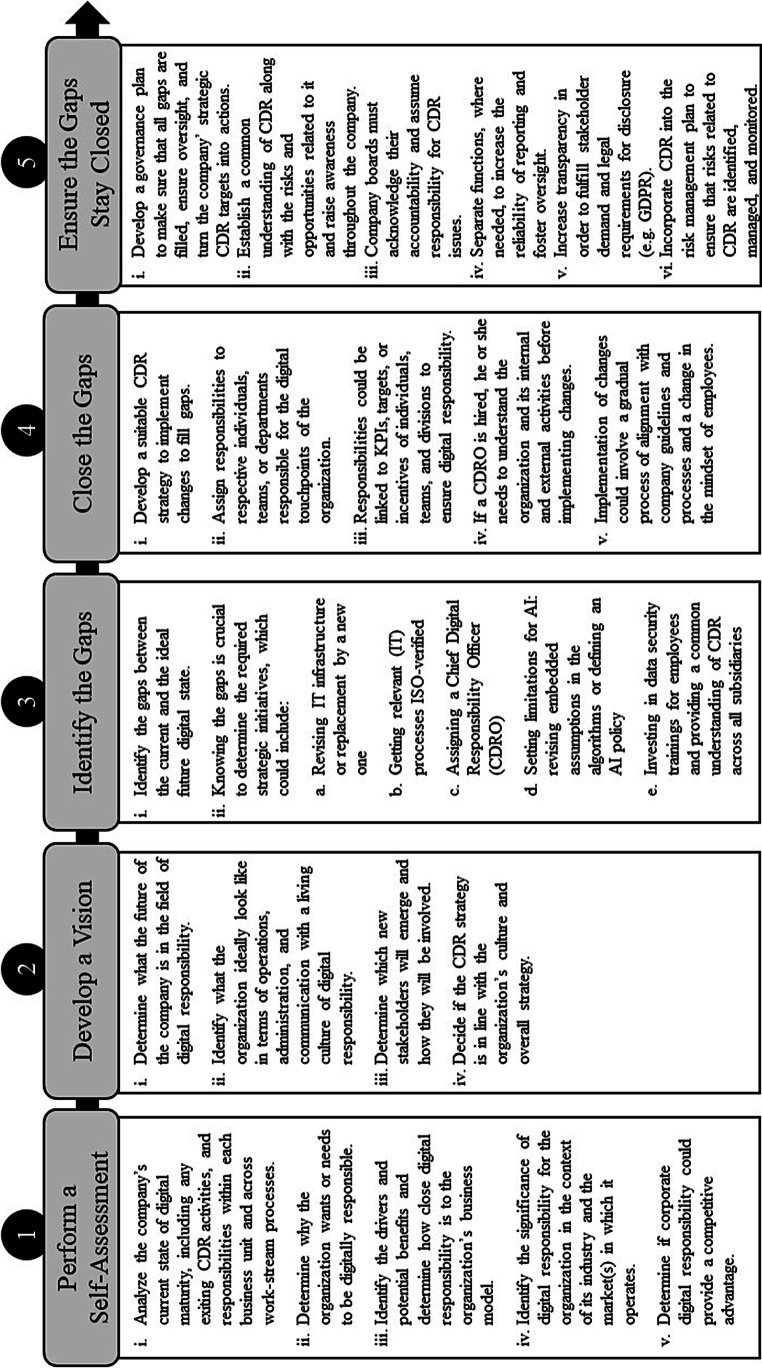
CSR Strategy Implementation Guide

The results of our review uncovered a need and an opportunity for companies to implement a CDR strategy to address the threats and embrace the opportunities of digitalization. As each company has unique goals, business strategies, and CDR needs, an individualized CDR strategy is essential. Using a gap analysis approach (e.g., Lin et al. 2009; Bunse et al. 2011), we propose the following five-step implementation guide for companies who intend to develop a CDR Strategy (see Fig. 5). Importantly, as the guide implies an understanding of the new corporate responsibilities in the digital age, new standards have to be integrated and brought to life in all value chain activities of the company. This view on CDR implementation resonates with Lobschat et al.’s (2019) understanding of CDR as a set of shared values and norms guiding the organization’s operations.

With technology accelerating at a lightning speed and new innovations continuously evolving in the digital space, companies should take an agile approach to CDR implementation and regularly revisit and update their current structures and CDR policies to ensure an optimum level of digital responsibility.

### Limitations and future research

The present paper aimed to define the concept of CDR by identifying and classifying CDR-related topics. Future research could build on this by assessing where certain topics have already been considered in other management tools or company divisions (e.g. in Bieser and Hilty 2018). Furthermore, specific indicators, instruments or methods that could be used to analyze and evaluate CDR-related topics could be identified and specific recommendations could be provided.

## Appendix

### Survey methodology

An online survey was sent to 509 US-based respondents to gain a deeper understanding of the prevailing public opinion on opportunities and threats of digitalization. To recruit respondents, Amazon’s Mechanical Turk (mturk) was used and respondents received 60 cents for taking part in the online survey which took less than 10 min. In terms of gender of the respondents, 53.8% self-identified as female, 44.8% as male, 0.6% as other and 0.8% preferred not to respond.

27.3% of the respondents stated to have a high school degree, 50.3% to have a Bachelors’ and 16.1% a Masters’ degree as their highest educational degree. Regarding participants’ sum of household incomes, 9.8% reported to have less than 1000 $US, 23.4% reported to have between 1001 $US and 2000 $US, 25.1% reported to have between 2001 $US and 3000 $US, and 41.7% reported a household income of more than 3001 $US. The average age in the sample was 36.8 years with a standard deviation of 11.9 years.

In the questionnaire, the participants were asked to rate their agreement to items on 7‑point Likert scale ranging from 1 = I do not agree at all to 7 = I fully agree. The items were based on the conceptual work on the topic areas of CDR. The questions were formulated as follows: for the opportunities, participants were asked to rate items such as “I think digitalization will provide many benefits such as … better services”. For the threats, participants were asked to rate items such as “I think digitalization will confront us with many challenges such as … cyber-crime” (Figs. 1 and 2 summarize the mean responses to these items).

Further, respondents were asked to assess the items “I have many *positive* thoughts and feelings related to digitalization” and “I have many *negative* thoughts and feelings related to digitalization”. The mean response is significantly higher for positive than for negative thoughts and feelings (mean_positive_ = 5.20, mean_negative_ = 3.84, *p* < 0.000).

Regarding the assessment of how well-prepared respondents feel for digitalization, a list of statements ranging form “I feel well-prepared for the digital transformation” to “I am scared that I could lose my job due to lack of digital skills” was provided. Participants were asked to rate their agreement to Likert-items on 7‑point rating scales ranging from 1 = I do not agree at all to 7 = I fully agree.

## Footnotes

1 A detailed description of the methodology is provided in the appendix.
